# A Pilot Study of the Effects of Mindfulness-Based Stress Reduction on Post-traumatic Stress Disorder Symptoms and Brain Response to Traumatic Reminders of Combat in Operation Enduring Freedom/Operation Iraqi Freedom Combat Veterans with Post-traumatic Stress Disorder

**DOI:** 10.3389/fpsyt.2017.00157

**Published:** 2017-08-25

**Authors:** James Douglas Bremner, Sanskriti Mishra, Carolina Campanella, Majid Shah, Nicole Kasher, Sarah Evans, Negar Fani, Amit Jasvant Shah, Collin Reiff, Lori L. Davis, Viola Vaccarino, James Carmody

**Affiliations:** ^1^Department of Psychiatry and Behavioral Sciences, Emory University, Atlanta, GA, United States; ^2^Department of Radiology, Emory University, Atlanta, GA, United States; ^3^Atlanta VA Medical Center, Decatur, GA, United States; ^4^Department of Psychiatry, University of Alabama, Birmingham, AL, United States; ^5^The Tuskegee VA Medical Center, Tuskegee, AL, United States; ^6^Department of Epidemiology, Rollins School of Public Health, Emory University, Atlanta, GA, United States; ^7^Department of Medicine, Division of Cardiology, Emory University School of Medicine, Emory University, Atlanta, GA, United States; ^8^Department of Medicine, Division of Preventive and Behavioral Medicine, University of Massachusetts Medical School, Worcester, MA, United States

**Keywords:** stress disorders, post-traumatic, brain imaging, positron-emission tomography, mindfulness training, mindfulness-based stress reduction, anterior cingulate cortex, insula

## Abstract

**Objective:**

Brain imaging studies in patients with post-traumatic stress disorder (PTSD) have implicated a circuitry of brain regions including the medial prefrontal cortex, amygdala, hippocampus, parietal cortex, and insula. Pharmacological treatment studies have shown a reversal of medial prefrontal deficits in response to traumatic reminders. Mindfulness-based stress reduction (MBSR) is a promising non-pharmacologic approach to the treatment of anxiety and pain disorders. The purpose of this study was to assess the effects of MBSR on PTSD symptoms and brain response to traumatic reminders measured with positron-emission tomography (PET) in Operation Enduring Freedom/Operation Iraqi Freedom (OEF/OIF) combat veterans with PTSD. We hypothesized that MBSR would show increased prefrontal response to stress and improved PTSD symptoms in veterans with PTSD.

**Method:**

Twenty-six OEF/OIF combat veterans with PTSD who had recently returned from a combat zone were block randomized to receive eight sessions of MBSR or present-centered group therapy (PCGT). PTSD patients underwent assessment of PTSD symptoms with the Clinician-Administered PTSD Scale (CAPS), mindfulness with the Five Factor Mindfulness Questionnaire (FFMQ) and brain imaging using PET in conjunction with exposure to neutral and Iraq combat-related slides and sound before and after treatment. Nine patients in the MBSR group and 8 in the PCGT group completed all study procedures.

**Results:**

Post-traumatic stress disorder patients treated with MBSR (but not PCGT) had an improvement in PTSD symptoms measured with the CAPS that persisted for 6 months after treatment. MBSR also resulted in an increase in mindfulness measured with the FFMQ. MBSR-treated patients had increased anterior cingulate and inferior parietal lobule and decreased insula and precuneus function in response to traumatic reminders compared to the PCGT group.

**Conclusion:**

This study shows that MBSR is a safe and effective treatment for PTSD. Furthermore, MBSR treatment is associated with changes in brain regions that have been implicated in PTSD and are involved in extinction of fear responses to traumatic memories as well as regulation of the stress response.

## Introduction

Post-traumatic stress disorder (PTSD) is an important clinical problem in populations exposed to traumatic experiences such as war, sexual assault, and natural disasters. Prevalence rates vary but large epidemiological studies show a lifetime prevalence of PTSD in the United States of about 8% (1). Increasing awareness and an increase in research in the field have furthered our understanding of this disorder, which is reflected in the revisions of the diagnostic criteria for PTSD and its reclassification from a form of anxiety disorder to its current place in trauma- and stressor-related disorders in the DSM-V criteria (2).

Operation Enduring Freedom/Operation Iraqi Freedom (OEF/OIF) entailed multiple deployments of American military personnel to Afghanistan and Iraq. Thirteen percent of soldiers deployed to Iraq were reported to have PTSD, and many of these soldiers did not seek mental health care (3–7). For those who do seek treatment, the available treatments are not always effective. Pharmacological treatment results in improvement in only a portion of PTSD patients and in others there is only a partial response to treatment (8). Psychological treatments involve either trauma-based and non-trauma-based interventions (9). Prolonged exposure therapy and cognitive processing therapy, two of the most well-studied forms of therapy, have been shown to be efficacious in many veterans with PTSD; however, about 30–60% of patients failed to show clinically significant improvement (10–12). Among the reasons cited for the same included high dropout rates of 30–40% due difficulties in tolerating the therapy, or failure to initiate therapy in the first place related to avoidance of traumatic reminders (13). These limitations have led to the search for alternative treatments for PTSD, such as mindfulness-based stress reduction (MBSR) and other mindfulness-based training approaches (14–16).

Mindfulness may be defined as intentionally paying attention to present-moment experience (physical sensations, affective states, thoughts, and imagery) in a non-judgmental way and thereby cultivating a stable and non-reactive awareness (17, 18).

Mindfulness is one of eight qualities cultivated in Buddhism to reduce psychic angst. It is the commonly used English translation of the word “sati” in the Pali cannon, where its meaning is akin to remembering awareness. In that system, relief comes through recognition of distress being created moment by moment through conditioned mental processes and behavior. It also teaches that everyday experience is composed of three phenomenological components; cognitions, sensations, and their pleasant/unpleasant feeling tones, which are interwoven in conditioned cycles of association. A thought gives rise to a feeling, that gives rise to a sensation, which reminds again of the thought, and on it goes.

Mindfulness training exercises are designed to cultivate recognition of these processes in real time. Attention is used to notice whichever thoughts, sensations, and feelings are appearing in awareness, while at the same time retaining the capacity to maintain the focus on these contents, or to redirect attention elsewhere. Recognition of this process, normally disguised beneath life’s everyday demands, results in a change in relationship with one’s thoughts, feelings, and sensations; rather than attention becoming preoccupied with their content, and trying to change or avoid them, they are recognized as events occurring in the field of awareness and will, by their nature, change. By providing a sense of control in stressful situations and encounters, this less-reactive response reduces the likelihood of being overwhelmed by thoughts and feelings and leaves mental space for more creative and less habitual responses.

Mindfulness training found its way into Western clinical settings largely through the work of Kabat-Zinn, who developed the manualized MBSR program (14). The program classes also provide education in applying those recognitions to habitual and reactive patterns in everyday situations as they are occurring (19–22). The program has been found to promote attention processes (23) and feelings of well-being, as well as reducing stress and worry (24–28). In the context of the present study, mindfulness training appears to target reactivity and experiential avoidance, key factors in the development and maintenance of PTSD (29). Because it is not based on processing the content of traumatic experience, it potentially circumvents problems related to treatment compliance related to symptoms of avoidance.

Mindfulness-based stress reduction has been shown to be effective for a variety of conditions, including chronic pain (14, 15, 30, 31), hot flashes (32), asthma (33), depression (16, 18, 20, 21, 34–38) fibromyalgia (39), psoriasis (40), and anxiety (14, 15, 17, 30, 41–44). MBSR is also helpful as an adjunctive intervention in patients with complex medical conditions, including promoting cancer recovery (16, 35), reducing psychological distress and depression, and promoting well-being and health-related quality of life (QOL), and sense of well-being (17, 18, 21, 36–38, 40, 41, 45–47) with positive effects persisting well beyond the initial treatment period (15, 47, 48).

Specific to PTSD, MBSR could potentially increase participants’ overall sense of control (39) through a positive accepting mode of control (49), which is associated with greater QOL and emotional well-being (50, 51). This less reactive mode of coping with difficulties may provide a way for PTSD patients to experience a greater sense of control in relation to their trauma-related thought and memories and to be less emotionally reactive to their presence (21, 52). This process may also prevent the rehearsing and replay of traumatic memories, which may modify the way they are stored and make them indelible, or resistant to further modification. A number of studies demonstrated the utility of MBSR for the promotion of well-being and mental health (14–17, 21, 30, 34–36, 40, 41, 45–48, 53–56).

Studies have assessed the effect of mindfulness-based programs on symptoms of PTSD (43, 57–62). An uncontrolled study in veterans with PTSD showed an improvement in PTSD with MBSR in comparison to baseline (59), A controlled study in veterans with PTSD of primary care brief mindfulness training showed a reduction in PTSD symptoms in comparison to treatment as usual (61). A controlled study of standard MBSR in veterans with PTSD, however, did not show clear improvements in PTSD symptoms compared to treatment as usual, although mental health QOL was improved, and there was a pattern of PTSD symptom response in treatment completers (58). Increases in mindfulness with MBSR, especially mindful awareness and non-reactivity, were associated with a decrease in PTSD symptoms when data from this study were combined with other data from the same group in a re-analysis (57). Another controlled study in veterans with PTSD showed reductions in self-reported PTSD symptoms with MBSR compared to present-centered group therapy (PCGT), but not clinician ratings as measured with the Clinician-Administered PTSD Scale (CAPS). Women with a history of interpersonal violence, of whom 38% met a checklist cutoff for PTSD, who underwent a trauma-informed MBSR treatment showed a reduction in symptoms compared to a wait-list control group (29). Patients with a history of childhood abuse, of whom 55% met a checklist cutoff for PTSD, who underwent an uncontrolled MBSR course of treatment showed a reduction in self-reported PTSD symptoms with treatment (60). In summary, no studies using active comparison groups have shown a reduction in PTSD symptoms with MBSR based on clinician ratings.

Previous studies have outlined a network of brain regions involved in symptoms of PTSD (63–65) that show functional responses to successful treatment (66). Brain imaging studies have identified a network of brain regions involved in the symptoms of PTSD that includes the amygdala, prefrontal cortex, hippocampus, parietal cortex, and insula (63), brain regions that play an important role in both memory, fear learning, and the physiological response to threat (67). The amygdala signals other parts of the brain to initiate the fight or flight response and in conjunction with hippocampus to convert this experience into a long-term memory. The suppression of the amygdala during extinction of fearful memories is mediated by the prefrontal cortex. The parietal cortex is involved in evaluating the self and other in space and time, which is an important facet of the stress response (68), and the insula serves as a gateway to peripheral responses to stress as well as regulating sense of time (69, 70). Positron-emission tomography (PET), single-photon emission computed tomography, and functional magnetic resonance imaging (fMRI) studies have shown traumatic reminders to result in decreased cerebral blood flow, and failure of activation in the medial prefrontal cortex (mPFC)/anterior cingulate in PTSD patients (63, 71–78). Other brain areas implicated in PTSD include the parietal cortex (79–83), precuneus (81, 84), and insula (84–90). These brain areas are involved memory, spatial attention, and coordination of peripheral physiological responses to stress.

A hypoactive frontal lobe leads to impairment in the differentiation of benign stresses from true stresses, consequently leading to reduction in the suppression of the amygdala manifesting as a hypervigilant state that is seen in PTSD (65). Fear learning paradigms have shown an increase in amygdala function in PTSD, and studies have shown both smaller hippocampal volume (91–93) and decreased function with memory tasks in PTSD patients. Smaller hippocampal volume and decreased medial prefrontal cortical function has been shown to reverse with both pharmacotherapy (94–99) and psychotherapies including eye movement desensitization reprocessing (100, 101) and cognitive-behavioral therapy (CBT) (102–105). Psychotherapy for PTSD also led to changes in parietal cortex function (79). Imaging studies suggest that MBSR may affect brain areas and physiological systems involved in the fear response in a beneficial way (31, 106–108). A recent study showed an increased connectivity between default mode network brain regions and frontal brain regions involved in executive control in PTSD patients treated with a novel therapy called mindfulness-based exposure therapy (109).

Studies have used functional brain imaging to study neural correlates of MBSR. MBSR has also been associated with changes in brain function and structure in normal individuals (110, 111). In social anxiety disorder patients, MBSR increased recruitment of posterior (parietal and occipital) cortical attention-related brain regions (111). No studies to date have assessed the effects of standard MBSR on PTSD. Furthermore, no studies have looked specifically at PTSD related to OIF/OEF. This is significant since studies in animals show that early intervention before memories become indelible may have a positive effect on the long-term course of PTSD (112). Understanding neural correlates of successful treatment response could improve understanding of the mechanisms by which successful treatments effects symptom improvement in the brain. This could be useful in guiding the development of new treatments. Brain imaging of neural correlates of response also provides an objective, quantifiable measure of treatment response that may be related to changes in the core pathophysiology.

The purpose of this study was to assess the efficacy of MBSR in the treatment of PTSD and the effects of MBSR on brain function in returning veterans with PTSD from deployment to combat in Iraq and Afghanistan. PTSD patients were randomized to 8 weeks of MBSR training or a control group that received 8 weeks of PCGT. PCGT involves weekly group sessions with a therapist that emphasize problem solving, being in the here and now, and health education. It was selected to control for the non-specific effects of attending weekly group sessions with other veterans with PTSD and participating in a treatment program. Subjects were assessed before and after treatment with brain imaging during exposure to neutral and combat-trauma-related slides and sounds. Brain imaging was performed with high-resolution positron-emission tomography (HR-PET) which is minimally invasive (requires insertion of an intravenous catheter) but offers superior anatomical resolution, a closer approximation to actual brain blood flow, and improved ease of presentation of visual and auditory materials compared to other imaging techniques such as fMRI. We hypothesized that MBSR (but not PCGT) would result in increased medial prefrontal and parietal cortex function and decreased insula and precuneus function with exposure to trauma-related stimuli.

## Materials and Methods

### Participants

Participants were male veterans aged 18–65 years returning from a combat deployment in Iraq or Afghanistan as part of OEF and OIF-OEF/OIF. All veterans had returned from deployment in the past year, had not been treated with psychotropic medications in the previous 4 weeks, and had the diagnosis of combat-related PTSD. All participants were recruited through fliers and public bulletins distributed within the community and the Mental Health Assessment Team at the Atlanta VA Medical Center. This study was approved by the Investigational Review Board of Emory University, and all enrolled subjects provided written informed consent. All participants spoke fluent English and had at least an eighth grade reading ability. Participants were considered ineligible if they had experienced significant head trauma or loss of consciousness for at least 2 min, or if they reported significant medical histories, current alcohol or substance abuse, or psychotic illness as identified by DSM-IV criteria in the Structured Clinical Interview for DSM-IV (SCID), or the diagnosis of PTSD preceding military service (113).

A total of 26 PTSD patients were found to be eligible and were randomly assigned in blocks to MBSR therapy (*N* = 17) and the control treatment, PCGT (*N* = 9). Nine subjects completed the MBSR group and eight the PCGT group as well as the other study procedures. Before attending the first class, three MBSR subjects dropped out because of lack of interest, and five because of lack of transportation and work-related issues. One member of the PCGT moved out of town before attending the first PCGT group. There were no differences in age, race, or years of education between the two groups (Table 1).

**Table 1:** Demographic factors in patients with post-traumatic stress disorder related to Operation Enduring Freedom/Operation Iraqi Freedom combat randomized to mindfulness based stress reduction (MBSR) and present-centered group therapy (PCGT).

	MBSR (*N* = 9)	PCGT (*N* = 8)
Mean age	34 (7 SD)	35 (10 SD)
Race	5 White, 4 African-American	5 White, 3 African-American
Years of education	16 (2)	15 (2)

### Treatment

All enrolled participants were randomized (by an outside researcher) by block to 8 weeks of treatment with MBSR or PCGT. The 8-week, 9-session MBSR intervention provides systematic and intensive training in mindfulness through formal meditation and mindful hatha yoga exercises, as well as application of their principles to everyday life and the range of challenges arising from real-life stressors and chronic diseases. In this way, participants were supported in becoming more aware of their internal resources and in acquiring skills that could be flexibly applied to cope more effectively with stress and PTSD symptoms. This range of self-regulatory skills were aimed at increasing relaxation and proprioceptive awareness, as well as awareness of the mind/body experiences related to their PTSD-related emotions and thoughts, overall sense of self, and self-in-relationship. Groups met for eight weekly, 2 1/2 h sessions and an all-day (6 h) session during week 6. The MBSR expert on the study team (JC) tailored the MBSR teaching materials to veterans, using metaphors that they could relate to, such as using analogies of attending to one’s breathing to training in sniper fire.

Formal mindfulness training was through: (a) body scan meditation, a gradual moving of attention through the body from feet to head accompanied by awareness of breathing and other bodily sensations while lying in a supine position, (b) sitting meditation, focusing on the awareness of breathing, bodily sensations, thoughts, and emotions, practiced sitting upright on a chair or cushion, and (c) mindful hatha yoga, stretching, and strengthening exercises practiced with awareness of breathing and intended to develop awareness (mindfulness) during movement. Participants were given two guided meditation CDs to be practiced at home for 30 min, 6 days/week. Subjects were assessed to determine the degree to which they followed homework assignments. In-class didactic material emphasized the systematic development of mindfulness awareness and its application in everyday life. Additional discussion focused on the psychology and physiology of stress reactivity and suggestions for the application of mindfulness as a method for responding positively to stress.

Mindfulness-based stress reduction instructors were trained by the Center for Mindfulness at the University of Massachusetts. All instructors and therapists did not participate in the research assessments. MBSR instructors were supervised by a research psychologist with expertise in MBSR (JC). Recordings were made of sessions which were reviewed by the supervisor to ensure adherence to treatment integrity.

In this study, PCGT was used as a control for the non-specific effects of a group-based intervention. PCGT was initially developed for use as a control group in a VA multisite study that tested the effects of trauma-focused group therapy in veterans with combat-related PTSD (no difference between the two groups was found for the treatment of PTSD) (114). The primary elements of PCGT include expectations of symptom reduction, normalization of PTSD symptoms though education, decreasing isolation, the opportunity to both give and receive support, and have positive experiences with others who also suffer from similar symptoms. PCGT also offers an atmosphere of safety and support where subjects can have an increased awareness of how PT SD affects their daily lives and those of other group members, and can gain more perspective and objectivity about the effects of PTSD on their lives. PCGT emphasized a “here-and-now” focus that avoided discussion of the trauma, as done in exposure therapy and cognitive restructuring. PCGT groups were conducted for the same number of hours as the MBSR groups. The initial phase of PCGT treatment was psycho-educational. After this phase, the primary content of PCGT was discussion of everyday problems of group members and coming to a better understanding of how PTSD creates or intensifies these problems. At the end of the program, there was a barbecue to balance the mindful retreat of the active group.

### Measures

The CAPS was used to assess PTSD symptoms at baseline and in response to treatment. The CAPS is a psychometrically sound measure that assesses presence and severity of PTSD (both lifetime and current PTSD) and can be used to assess changes in PTSD symptoms with treatment (115). The CAPS includes indices measuring reexperiencing, avoidance and numbing, and hyperarousal symptoms. The CAPS has a coefficient alpha of 0.94, test–retest reliability of 0.9–0.98, a sensitivity of 0.91, and specificity of 0.86 for diagnosis of PTSD, and good convergent and divergent validity (115, 116).

Structured Clinical Interview for DSM-IV (with PTSD module) was used to establish psychiatric diagnosis. The PTSD module of the SCID has been shown to have a kappa of 0.93 for test–retest reliability, and excellent sensitivity (0.81) and specificity (0.98) against a composite PTSD diagnosis (117).

Mindfulness was assessed with the Five Factor Mindfulness Questionnaire (FFMQ), a validated measure of the capacity for mindfulness (118). This instrument was derived from a factor analysis of questionnaires measuring a trait-like general tendency to be mindful in daily life. It consists of 39 items assessing five facets of mindfulness: observing, describing, acting with awareness, non-judging of inner experience, and non-reactivity to inner experience. Items are rated on a Likert scale ranging from 1 (never or very rarely true) to 5 (very often or always true). The FFMQ has been shown to have good internal consistency and significant relationships in the predicted directions with a variety of constructs related to mindfulness (118, 119).

Mindful spirituality was assessed with the Functional Assessment of Chronic Illness Therapy-Spiritual Well-Being Scale (FACIT-Sp), a valid and reliable 12-item scale designed to measure spiritual well-being independent of religious beliefs (120). It has been adapted for use with non-medical populations and comprises two subscales: Meaning and Peace (8 items) and Faith (4 items) (121). The FACIT-Sp (120) is part of the FACIT battery (122) to assess QOL associated with chronic illness.

### PET Procedures

High-resolution positron-emission tomography scanning was conducted using methods previously described (94). HR-PET imaging of the brain was performed with the high-resolution research tomograph (CTI, Knoxville, TN, USA) (<2 mm resolution) (123, 124). Subjects were scanned during exposure to traumatic (Iraqi combat) slides, and sounds, and neutral slides and sounds using methods previously described (125). Subjects underwent four scans in conjunction with injection of radiolabeled water: two successive scans while watching neutral slides and sounds, followed by two scans while viewing combat-related slides and sounds. Content of the slides was matched between combat and neutral for sound and content, e.g., scenes involving persons and buildings in Iraq with loud noises were matched with a city scene with buildings and persons and city traffic noises. Radiolabeled water (H_2_[^15^O]) was prepared on-site in a cyclotron. During the hours of the test, subjects reclined in bed except to use the bathroom. An intravenous infusion of normal saline was started by a technologist to permit the bolus injection of radiolabeled water. Subjects were scanned with eyes open in a dimly lit room. Subjects were placed in the scanner with the head held in a head holder, and the head was positioned with the canthomeatal line parallel to the external laser light. Following positioning within the camera gantry, a transmission scan of the head was obtained. Subjects received a 20 mCi intravenous bolus of radiolabeled water for each of the scans. Subjects were scanned twice during exposure to neutral slides and sounds (city scenes with people, buildings, city sounds, people talking, birds tweeting), and twice during exposure to Iraq combat scenes and sounds. Each scan lasted 60 s, with the PET scan acquisition beginning at the initiation of the condition.

### Statistical Analysis

Post-traumatic stress disorder symptoms as measured with the CAPS were compared before and after intervention with MBSR or PCGT using one-tailed *t*-tests with significance defined as *p* < 0.05. HR-PET images were reconstructed and analyzed using Statistical Parametric Mapping software (SPM8) (126, 127). Regions of interest were identified within hypothesized brain regions (mPFC, parietal cortex, insula, precuneus) using standard stereotactic coordinates (128). Images were realigned to the first scan of the study session. The mean concentration of radioactivity in each scan was obtained as an area-weighted sum of the concentration of each slice, and was adjusted to a nominal value of 50 ml/min per 100 g. The data underwent transformation into a common anatomical space and were smoothed with a three-dimensional Gaussian filter to 8-mm FWHM. Regional blood flow was compared for traumatic minus neutral slides, and sounds conditions. Statistical analyses yielded image data sets in which the values assigned to individual voxels correspond to the *t* statistic. Statistical images were displayed with values of *z* score units. Location of areas of activation was identified as the distance from the anterior commissure in millimeters, with *x, y*, and *z* coordinates; a standard stereotaxic (Talairach) atlas was used (128). A threshold *z* score of 2.67 (*p* < 0.005, uncorrected) and a minimal cluster of 11 voxels was used to examine areas of activation within hypothesized areas (medial prefrontal and parietal cortex, precuneus, insula) (127, 129, 130). This level of significance has been shown to optimize the reduction of risk of both Type 1 and Type 2 errors (131, 132). Activations in non-hypothesized regions meeting this statistical threshold were also presented for the purpose of hypothesis generation for future studies.

## Results

**Figure 1:**
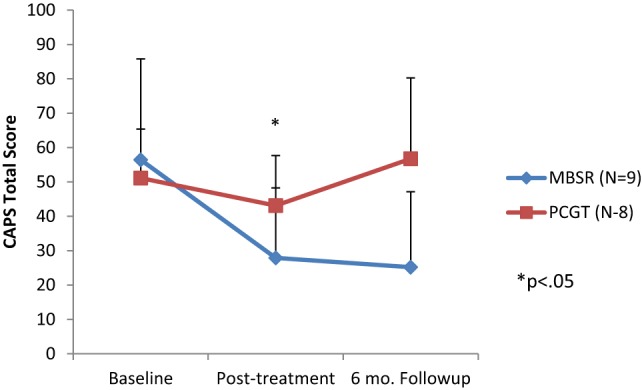
Effects of 8 weeks of mindfulness-based stress reduction (MBSR), or present-centered group therapy (PCGT) on symptoms of post-traumatic stress disorder (PTSD) as measured with the Clinician-Administered PTSD Scale (CAPS). There was a significant reduction in PTSD symptoms immediately posttreatment in the MBSR group (*p* < 0.05), but not in the PCGT group. These effects persisted at the 6-month follow-up period.

There were no differences in demographic variables between PTSD patients randomized to either MBSR or PCGT (Table 1). Eight weeks of MBSR therapy (but not PCGT) resulted in significant reductions in PTSD symptoms (Figure 1; Table 2). These effects persisted 6 months after the ends of treatment (Figure 1). The greatest reductions in symptoms were seen in the CAPS hyperarousal symptom cluster in the MBSR group [31 (14 SD) baseline versus 16 (11 SD)] (*p* < 0.006) although significant changes were seen in the avoidance (but not intrusion) cluster as well. MBSR also resulted in an increase in mindfulness as measured by the FFMQ (Table 2) as well as a non-statistically significant increase of 23% in spiritual mindfulness as measured by the FACIT-Sp (*p* = 0.06; Table 2). Increases were equally seen in the Faith and Meaning/Purpose Subscales.

**Table 2:** Effects of MBSR and PCGT on symptoms of post-traumatic stress disorder and measures of mindfulness and spirituality.

Measure	Pre-MBSR	Post-MBSR	Pre-PCGT	Post-PCGT	*p*-Value*
CAPS-I	10 (9 SD)	3 (4 SD)	7 (8 SD)	8 (5 SD)	0.14
CAPS-Av	16 (9 SD)	8 (7 SD)	13 (6 SD)	8 (4 SD)	0.026
CAPS-Ar	31 (14 SD)	16 (11 SD)	32 (6 SD)	27 (8 SD)	0.006
CAPS-TOTAL	56 (29 SD)	28 (20 SD)	51 (14 SD)	43 (15 SD)	0.016
FFMQ	121 (11 SD)	139 (22 SD)	121 (19 SD)	127 (23 SD)	0.04
FACIT-Sp	52 (18 SD)	64 (21 SD)	58 (16 SD)	61 (18 SD)	0.06

*CAPS-I, CAPS-Intrusions Subscale; CAPS-Av, CAPS-Avoidance Subscale; CAPS-Ar, CAPS-Arousal Subscale; CAPS-TOTAL, CAPS total score; FFMQ, Five Factor Mindfulness Scale; FACIT-Sp, Functional Assessment of Cancer Illness Therapy-Spirituality Subscale; PCGT, present-centered group therapy; MBSR, mindfulness-based stress reduction*.

*p-Value for difference between outcome before and after MBSR therapy.

**Figure 2:**
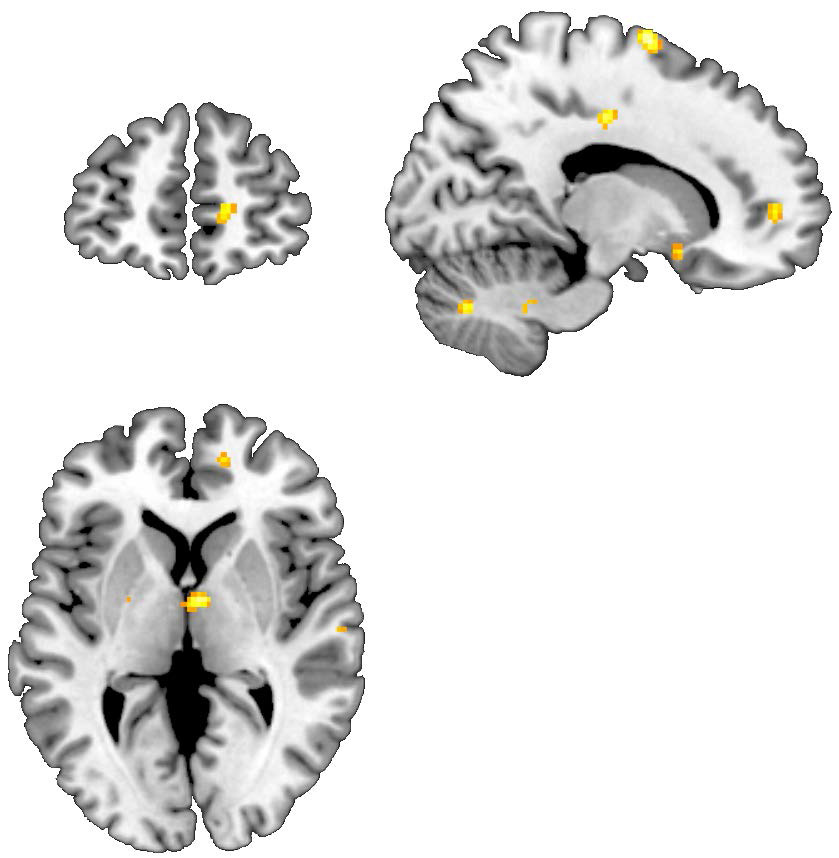
Areas of increased activation when viewing traumatic pictures, and sounds vs. neutral pictures and sounds, Mindfulness-based stress reduction > traditional supportive therapy. Increased activations in anterior cingulate, and subcallosal gyrus.

Exposure to combat-related slides, and sounds resulted in increased activation in multiple frontal, and temporal cortical brain regions in both the MBSR and PCGT-treated groups (Tables 3–6). Both groups showed additionally decreased activation in subcortical areas, insula, and cerebellum. PTSD patients treated with MBSR also showed a differential brain response to combat-trauma-related slides, and sounds when compared to the PCGT group, with an increased activation in the right anterior cingulate, and right inferior parietal lobule (Figure 2; Table 7), and decreased activation in the right insula and precuneus (Table 8).

**Table 3:** Areas of increased activation for traumatic slides and sounds after present-centered group therapy treatment.

		Talairach coordinates				
*z* Score	Voxel number	*x*	*y*	*z*	Brain region	Brodmann’s area
3.49	41	−8	13	62	L. superior frontal gyrus	6
3.37	30	−6	31	46	L. superior frontal gyrus	8
3.15	24	24	50	27	R. superior frontal gyrus	10
2.92	14	−16	52	21	L. superior frontal gyrus	10
2.82		−16	49	12	L. medial frontal gyrus	10
2.89	17	20	30	48	R. superior frontal gyrus	8
3.68	22	38	42	−7	R. middle frontal gyrus	11
2.97	15	40	18	41	R. middle frontal gyrus	8
3.39	21	−34	58	4	L. middle frontal gyrus	10
3.18	15	−32	14	40	L. middle frontal gyrus	8
3.16	14	−42	6	44	L. middle frontal gyrus	8
3.43	57	−48	25	−8	L. inferior frontal gyrus	47
3.33		−40	23	−8	L. inferior frontal gyrus	47
3.04		−48	32	−15	L. inferior frontal gyrus	47
2.98	15	42	51	1	R. inferior frontal gyrus	10
4.07	33	−6	48	27	L. medial frontal gyrus	9
3.71	36	−2	16	45	L. medial frontal gyrus	6
4.44	69	32	−20	64	R. precentral gyrus	4
3.48	12	−40	−15	50	L. precentral gyrus	4
3.45	21	46	−18	38	R. precentral gyrus	4
3.67	26	−6	51	−21	L. orbital gyrus	11
3.31	19	0	−37	28	Bilateral cingulate gyrus	31
3.12	20	−12	12	5	L. caudate	
3.52	22	−28	−18	−2	L. putamen	
3.06	13	−44	8	−29	L. middle temporal gyrus	21
2.99	12	61	−53	−11	R. inferior temporal gyrus	20
3.16	19	28	−72	33	R. precuneus	19

*z > 2.68, p < 0.005*.

**Table 4:** Areas of decreased activations for traumatic slide and sounds after present-centered group therapy treatment.

		Talairach coordinates				
*z* Score	Voxel number	*x*	*y*	*z*	Brain region	Brodmann’s area
2.93	14	12	5	64	R. superior frontal gyrus	6
3.20	16	38	44	24	R. middle frontal gyrus	10
3.54	37	−55	7	14	L. inferior frontal gyrus	44
3.65	100	59	10	9	R. precentral gyrus	44
3.27		51	10	8	R. precentral gyrus	44
3.47	11	−34	−42	56	L. postcentral gyrus	5
3.22	13	55	−24	16	R. postcentral gyrus	40
3.85	29	12	−12	41	R. cingulate gyrus	31
3.48	23	6	−2	30	R. cingulate gyrus	24
3.40	18	−26	6	7	L. putamen	
3.08	22	−26	−4	−3	L. putamen	
3.46	20	10	11	10	R. caudate	
3.49	17	−30	−19	12	L. claustrum	
3.24	13	26	12	9	R. claustrum	
3.35	52	6	−4	4	R. thalamus	
3.19	11	−26	−31	7	L. thalamus	
2.92	29	−44	−36	22	L. insula	13
3.20	26	57	−15	6	R. superior temporal gyrus	41
2.99	23	67	−32	22	R. superior temporal gyrus	42
3.41	14	−44	−12	−10	L. middle temporal gyrus	21
3.34	17	63	−30	−24	R. inferior temporal gyrus	20
3.27	23	−55	−37	39	L. inferior parietal lobule	40
3.05	15	0	−83	41	Bilateral precuneus	7
3.22	11	−16	−44	52	L. precuneus	7
3.43	37	−6	−92	30	L. cuneus	19
3.26		−2	−90	23	L. cuneus	18
4.80	106	−30	−52	−42	L. cerebellum	
3.83	63	28	−60	−50	R. cerebellum	
2.67		24	−68	−57	R. cerebellum	
3.60	165	40	−46	−37	R. cerebellum	
3.43		36	−56	−45	R. cerebellum	
3.33		36	−62	−50	R. cerebellum	
3.38	21	18	−48	−39	R. cerebellum	
3.32	12	−26	−73	−60	L. cerebellum	
3.31	26	16	−51	−40	R. cerebellum	
3.21		6	−49	−38	R. cerebellum	
3.25	21	−40	−46	−37	L. cerebellum	
3.18	19	44	−44	−35	R. cerebellum	
3.17	13	−14	−48	−39	L. cerebellum	
3.14	23	−36	−65	−53	L. cerebellum	

*z > 2.68, p < 0.005*.

**Table 5:** Areas of increased activation for traumatic slides and sounds after mindfulness-based stress reduction treatment.

		Talairach coordinates				
*z* Score	Voxel number	*x*	*y*	*z*	Brain region	Brodmann’s area
3.75	33	16	3	68	R. superior frontal gyrus	6
3.51	29	−10	44	35	L. superior frontal gyrus	8
3.44	36	22	54	21	R. superior frontal gyrus	10
3.38	62	−24	58	3	L. superior frontal gyrus	10
3.04		−26	62	−5	L. superior frontal gyrus	10
3.30	28	18	22	56	R. superior frontal gyrus	6
2.75		10	16	56	R. superior frontal gyrus	6
3.22	20	−10	59	−18	L. superior frontal gyrus	11
3.20	39	10	32	52	R. superior frontal gyrus	6
3.07		0	33	48	Bilateral superior frontal gyrus	8
3.05	23	2	5	55	R. superior frontal gyrus	6
2.80		10	11	60	R. superior frontal gyrus	6
3.29	39	28	−3	52	R. middle frontal gyrus	6
3.04	14	−34	14	42	L. middle frontal gyrus	6
3.00	13	−40	34	−12	L. middle frontal gyrus	11
3.30	16	46	48	−2	R. inferior frontal gyrus	10
3.08	21	30	13	−9	R. inferior frontal gyrus	13
3.02	37	−38	20	−18	L. inferior frontal gyrus	47
2.97	11	−28	25	−3	L. inferior frontal gyrus	47
2.95	11	46	27	−11	R. inferior frontal gyrus	47
2.90	24	−36	−4	−37	L. inferior temporal gyrus	20
2.84		−46	−2	−35	L. inferior temporal gyrus	20
4.18	133	14	55	5	R. medial frontal gyrus	10
3.82		18	64	6	R. medial frontal gyrus	10
3.53	35	6	49	14	R. medial frontal gyrus	9
3.36	191	8	35	35	R. medial frontal gyrus	8
3.18		4	49	38	R. medial frontal gyrus	8
3.15		18	43	38	R. superior frontal gyrus	9
2.87	12	−4	50	21	L. medial frontal gyrus	9
4.11	94	−28	−26	60	L. precentral gyrus	4
2.91		−34	−21	49	L. precentral gyrus	4
3.22	13	−34	21	36	L. precentral gyrus	9
3.06	38	−36	0	37	L. precentral gyrus	6
3.04		−36	−6	43	L. middle frontal gyrus	6
3.82	59	10	−30	68	R. postcentral gyrus	3
3.14	12	−10	−31	70	L. postcentral gyrus	3
3.77	50	2	46	−21	R. orbital gyrus	11
3.59	18	−6	10	36	L. cingulate gyrus	32
3.57	13	−22	−62	9	L. posterior cingulate	30
3.07	30	10	−41	32	R. cingulate gyrus	31
2.85		6	−35	35	R. cingulate gyrus	31
3.05	27	−16	21	38	L. cingulate gyrus	32
3.04	14	2	38	15	R. anterior cingulate	32
3.31	12	−12	−26	14	L. thalamus	
3.28	40	2	−6	4	R. thalamus	
2.98	11	−24	−8	0	L. lateral globus pallidus	
3.07	17	−28	13	−4	L. claustrum	
3.40	15	−34	−15	−28	L. uncus	20
3.51	51	34	18	−23	R. superior temporal gyrus	38
2.74		28	28	−20	R. inferior frontal gyrus	11
3.42	23	−53	−71	16	L. middle temporal gyrus	19
2.80	11	61	−37	−10	R. middle temporal gyrus	21
3.86	108	46	−11	−31	R. inferior temporal gyrus	20
3.14		36	−19	−28	R. uncus	20
3.21	25	51	−2	−37	R. inferior temporal gyrus	20
3.02		46	8	−37	R. middle temporal gyrus	38
3.42	27	−22	−53	63	L. superior parietal lobule	7
3.38	28	32	−66	48	R. superior parietal lobule	7
3.99	99	50	−60	40	R. inferior parietal lobule	39
2.87		46	−52	43	R. inferior parietal lobule	40
3.38	22	44	−70	37	R. precuneus	39
3.85	186	−2	−55	36	L. precuneus	7
3.56		−2	−47	37	L. cingulate gyrus	31
3.24		2	−64	31	R. cuneus	7
3.77	26	−4	−69	51	L. precuneus	7
3.65	37	4	−51	−40	R. cerebellum	
3.38	11	55	−61	−20	R. cerebellum	
3.00	13	14	−69	−25	R. cerebellum	
2.97	15	34	−83	−23	R. cerebellum	

*z > 2.68, p < 0.005*.

**Table 6:** Areas of decreased activation for traumatic slides and sounds after mindfulness-based stress reduction treatment.

		Talairach coordinates				
*z* Score	Voxel number	*x*	*y*	*z*	Brain region	Brodmann’s area
4.10	272	61	−10	26	R. precentral gyrus	4
3.39	17	51	−4	37	R. precentral gyrus	6
2.97	19	−48	−5	8	L. precentral gyrus	6
4.17	103	−61	−15	10	L. transverse temporal gyrus	42
2.94		−63	−9	19	L. postcentral gyrus	43
2.76		−59	−3	13	L. precentral gyrus	4
3.43	17	55	−21	10	R. transverse temporal gyrus	41
4.21	25	−42	−11	19	L. insula	13
3.50	106	42	3	13	R. insula	13
3.46		42	−15	12	R. insula	13
3.16		34	−19	8	R. claustrum	
3.00	13	40	−4	−1	R. insula	13
3.46	30	−28	−3	13	L. claustrum	
2.83		−36	−10	4	L. claustrum	
3.37	51	38	−14	−8	R. caudate	
3.08		48	−6	−6	R. superior temporal gyrus	22
2.72		40	−4	−8	R. middle temporal gyrus	21
3.32	16	48	−21	5	R. superior temporal gyrus	41
3.08	24	63	−7	8	R. superior temporal gyrus	22
3.29	15	−51	7	−15	L. middle temporal gyrus	21
3.04	13	67	−50	8	R. middle temporal gyrus	21
3.21	15	−4	−97	−7	L. lingual gyrus	18
3.74	24	−8	−28	−9	L. cerebellum	
3.38	18	32	−44	−20	R. cerebellum	
3.32	48	16	−29	−29	L. cerebellum	
2.95		20	−32	−22	R. cerebellum	
3.23	18	−26	−44	−20	L. cerebellum	
3.17	11	−2	−73	−30	L. cerebellum	
3.08	12	40	−50	−36	R. cerebellum	
3.42	13	−6	−31	−35	L. medulla	

*z > 2.68, p < 0.005*.

**Table 7:** Areas of increased activation for traumatic slides and sounds in mindfulness-based stress reduction greater than present-centered group therapy.

		Talairach coordinates				
*z* Score	Voxel number	*x*	*y*	*z*	Brain region	Brodmann’s area
3.21	18	14	5	68	R. superior frontal gyrus	6
3.23	15	−36	30	19	L. middle frontal gyrus	46
2.97	14	22	14	56	R. middle frontal gyrus	6
3.25	11	−32	−19	49	L. precentral gyrus	4
3.24	11	59	−6	43	R. precentral gyrus	6
3.07	12	10	−24	29	R. cingulate gyrus	23
2.83	12	2	−2	35	R. cingulate gyrus	24
3.05	11	−22	−6	−1	L. lateral globus pallidus	
3.05	14	26	13	−6	R. claustrum	
3.13	19	−8	−16	−3	L. subthalamic nucleus	
3.40	21	59	−13	6	R. superior temporal gyrus	22
3.37	71	53	−37	41	R. inferior parietal lobule	40
3.29	16	34	−53	38	R. inferior parietal lobule	40
2.90	11	55	−48	47	R. inferior parietal lobule	40
3.21	50	42	−68	35	R. precuneus	39
3.68	66	−26	−69	−22	L. cerebellum	
3.12		−28	−62	−29	L. cerebellum	
3.62	25	36	−2	−10	R. cerebellum	
3.36	44	46	−61	−20	R. cerebellum	
3.31		36	−57	−21	R. cerebellum	
2.82	15	18	−68	−32	R. cerebellum	
2.90	12	−6	−23	−32	L. pons	

*z > 2.68, p < 0.005*.

**Table 8:** Areas of decreased activation for traumatic slides and sounds in mindfulness-based stress reduction greater than present-centered group therapy.

		Talairach coordinates				
*z* Score	Voxel number	*x*	*y*	*z*	Brain region	Brodmann’s area
3.1669	23	61	−10	26	R. precentral gyrus	4
3.1252	33	−59	−15	8	L. transverse temporal gyrus	42
2.8132	17	42	−7	10	R. insula	13
3.1287	17	4	−99	9	R. cuneus	18

*z > 2.68, p < 0.005*.

## Discussion

Patients with PTSD related to Iraq combat treated with MBSR in this study had a decrease in PTSD symptoms as measured with the CAPS not seen in PTSD patients treated with the control condition, PCGT. PTSD patients treated with MBSR also showed a differential brain response to combat-trauma-related slides, and sounds when compared to the PCGT group, with an increased activation in the right anterior cingulate cortex (ACC) and right inferior parietal lobule, and decreased activation in the right insula, and precuneus. MBSR resulted in an increase in mindfulness as measured with the FFMQ. Results from a measure of spirituality showed a 23% improvement that was not statistically significant.

Mindfulness-based stress reduction was well tolerated in this sample of veterans with PTSD. Although there are several reports of the use of treatments similar to MBSR now in the literature, at the time of this study there was little experience with MBSR in traumatized populations. MBSR in this sample of PTSD patients did not lead to flooding of traumatic memories, dissociation, or other negative consequences. Additionally, MBSR treatment had a robust effect on PTSD symptoms, leading to reductions that maintained for 6 months after the end of the classes. This is consistent with sustained (12 months) improvements in distress found in an RCT comparing MBSR to an educational control program (33). It was also superior to PCGT, which has been shown to have some efficacy in and of itself, showing equal efficacy to Cognitive-Behavioral Group Therapy (CBGT) in a prior multisite trial in veterans (114).

This study suggests that treatment with MBSR results in increased function in the anterior cingulate cortex (ACC) region within the mPFC. Prior studies from our group, and others have implicated this area in the pathophysiology of PTSD (63). Additionally, we previously found that successful treatment with the selective serotonin reuptake, paroxetine, in PTSD patients resulted in an increase in function in this area in response to trauma-related scripts of the patients’ personal individual traumas (94). Activation was also seen in this area with placebo, although to a lesser degree than with paroxetine (94). In contrast, there were no activations in this area with the PCGT control treatment in the current study.

The ACC plays an important role in emotion, and regulation of the fear response (133, 134). It has connections to limbic areas that mediate threat response and extinction of conditioned fear responses that are characteristic of PTSD (135) as well as with the insula in the regulation of peripheral autonomic and neurohormonal responses to stress (69, 133, 134, 136). Exposure to traumatic reminders has been shown in a number of studies of PTSD patients to result in a blunted ACC activation (74, 76, 77, 83, 137, 138). Training with MBSR may enhance the ability of the ACC to inhibit fear responses that occur in response to traumatic reminders. Indeed, uncontrolled fear responses to traumatic reminders are a paradigmatic facet of the disorder of PTSD (139).

Mindfulness-based stress reduction also resulted in an increase in function in the inferior parietal lobule. This brain area plays an important role in the perception of the self in time and space, perception of contextual cues, and visuospatial memory (140–143), in addition to modulation of peripheral cardiovascular responses to stress (144–146). Prior studies also implicated this region in PTSD (79). Increased function in this area with MBSR training may be indicative of an enhancement of the ability to successfully respond to threat.

Mindfulness-based stress reduction also resulted in a decrease in function in the insula and precuneus. The precuneus has important connections with the parietal cortex and is involved in memory, which is an important part of the stress response (147, 148). Prior studies in PTSD have implicated dysfunction of a network that includes precuneus, anterior cingulate, and insula (84).

The insula is an important portal to peripheral responses to stress, including cardiovascular responses such as heart rate and blood pressure, respiratory function like increased respirations, and other functions important to survival under stress (69, 145). It also has important connections to brain areas involved in the stress response (70), and altered function and structure in this region has been linked to PTSD (70, 84, 86, 137, 149–153).

Decreased function with MBSR may represent a decrease in peripheral stress response to traumatic reminders.

Changes in brain function with MBSR appeared to be localized to the right side of the brain. The right side of the brain is more involved in emotion, while the left side in logical thought (82, 154, 155). Although suggestive that MBSR facilitates emotion-related brain areas, this finding needs to be replicated.

There are several limitations of this study that should be noted. Our findings are in patients with OIF/OEF combat-related PTSD and cannot necessarily be generalized to other PTSD patient populations. The sample size is small, which limited the statistical analyses, and therefore this should be considered a pilot study. Due to the small sample size, we were not able to adjust for potentially confounding factors such as differences in PTSD symptom severity. These pilot findings need to be replicated in larger, controlled studies. This is the first study, however, to use brain imaging in the assessment of the effects of MBSR in PTSD, and should generate useful hypotheses for future studies with larger sample sizes.

In summary, we found that MBSR results in an improvement in PTSD symptoms, an increase in mindfulness that could be associated with an improvement in spiritual well-being, and a pattern of brain activation to traumatic reminders that included increased function in anterior cingulate, and inferior parietal lobe, and decreased function in insula and precuneus. These brain areas are involved in stress, and the fear response. The findings suggest that MBSR leads to a change in brain function that is associated with a decrease in activation of fear, and stress responses in PTSD patients.

## Ethics Statement

This study was approved by the Investigational Review Board of Emory University, and all enrolled subjects provided written informed consent.

## Author Contributions

All authors designed the study, obtained funding, supervised and trained teachers, monitored treatment fidelity, and contributed to the editing of the paper.

## Conflict of Interest Statement

The authors declare that the research was conducted in the absence of any commercial or financial relationships that could be construed as a potential conflict of interest.
